# A genome-wide SNP-association study confirms a sequence variant (g.66493737C>T) in the equine myostatin (*MSTN*) gene as the most powerful predictor of optimum racing distance for Thoroughbred racehorses

**DOI:** 10.1186/1471-2164-11-552

**Published:** 2010-10-11

**Authors:** Emmeline W Hill, Beatrice A McGivney, Jingjing Gu, Ronan Whiston, David E MacHugh

**Affiliations:** 1 Animal Genomics Laboratory, School of Agriculture, Food Science and Veterinary Medicine, University College Dublin, Belfield, Dublin 4, Ireland; 2 Equinome Ltd., NovaUCD, Belfield Innovation Park, Belfield, Dublin 4, Ireland; 3 Conway Institute of Biomolecular and Biomedical Research, University College Dublin, Belfield, Dublin 4, Ireland

## Abstract

**Background:**

Thoroughbred horses have been selected for traits contributing to speed and stamina for centuries. It is widely recognized that inherited variation in physical and physiological characteristics is responsible for variation in individual aptitude for race distance, and that muscle phenotypes in particular are important.

**Results:**

A genome-wide SNP-association study for optimum racing distance was performed using the EquineSNP50 Bead Chip genotyping array in a cohort of *n *= 118 elite Thoroughbred racehorses divergent for race distance aptitude. In a cohort-based association test we evaluated genotypic variation at 40,977 SNPs between horses suited to short distance (≤ 8 f) and middle-long distance (> 8 f) races. The most significant SNP was located on chromosome 18: BIEC2-417495 ~690 kb from the gene encoding myostatin (*MSTN*) [*P*_unadj. _= 6.96 × 10^-6^]. Considering best race distance as a quantitative phenotype, a peak of association on chromosome 18 (chr18:65809482-67545806) comprising eight SNPs encompassing a 1.7 Mb region was observed. Again, similar to the cohort-based analysis, the most significant SNP was BIEC2-417495 (*P*_unadj. _= 1.61 × 10^-9^; *P*_Bonf. _= 6.58 × 10^-5^). In a candidate gene study we have previously reported a SNP (g.66493737C>T) in *MSTN *associated with best race distance in Thoroughbreds; however, its functional and genome-wide relevance were uncertain. Additional re-sequencing in the flanking regions of the *MSTN *gene revealed four novel 3' UTR SNPs and a 227 bp SINE insertion polymorphism in the 5' UTR promoter sequence. Linkage disequilibrium was highest between g.66493737C>T and BIEC2-417495 (*r*^2 ^= 0.86).

**Conclusions:**

Comparative association tests consistently demonstrated the g.66493737C>T SNP as the superior variant in the prediction of distance aptitude in racehorses (g.66493737C>T, *P *= 1.02 × 10^-10^; BIEC2-417495, *P*_unadj. _= 1.61 × 10^-9^). Functional investigations will be required to determine whether this polymorphism affects putative transcription-factor binding and gives rise to variation in gene and protein expression. Nonetheless, this study demonstrates that the g.66493737C>T SNP provides the most powerful genetic marker for prediction of race distance aptitude in Thoroughbreds.

## Background

Thoroughbred horses have been selected for structural and functional variation contributing to speed and stamina during the three century development of the breed. The International Federation of Horseracing Authorities recognizes five distance categories: Sprint (5-6.5 furlongs [f], ≤ 1,300 m), Mile (6.51-9.49 f, 1,301-1,900 m), Intermediate (9.5-10.5 f, 1,901-2,112 m), Long (10.51-13.5 f, 2,114-2,716 m) and Extended (> 13.51 f, > 2,717 m) races (http://www.horseracingintfed.com) and it is widely recognized among horse breeders that variation in physical and physiological characteristics are responsible for variation in individual aptitude for race distance [1]. Although environment and training may contribute to the race distance for which a horse is best suited, the genetic contribution to the ability to perform optimally at certain distances is large; the heritability of best distance among Australian racehorses has been estimated as 0.94 ± 0.03 [2].

A principal characteristic contributing to the ability of a Thoroughbred to perform well in short distance, sprint races is the extent and maturity of the skeletal musculature. Sprinters are generally shorter, stockier animals with greater muscle mass than animals suited to endurance performance, and generally mature earlier. Performance aptitude for speed and stamina has also been associated with muscle fibre type phenotypes [3,4] and metabolic adaptations to training [5]. Variation in cardiovascular function contributing to aerobic capacity may also play a role in distinguishing individuals suited to shorter or longer distance races.

We have previously reported a sequence polymorphism (g.66493737C>T) in the equine myostatin (*MSTN*) gene strongly associated (*P = *4.85 × 10^-8^) with optimum racing distance in Thoroughbred racehorses [6]. In several mammalian species, including cattle, sheep, dogs and horses, muscle hypertrophy phenotypes are associated with sequence variants in the *MSTN *gene [7-11]. Among horses that compete preferably in short distance (≤ 7 f) races requiring exceptional speed, the C allele is twice as common than among horses that perform optimally in longer distance (> 8 f) races that require more stamina (0.72 and 0.36 respectively). On average the optimum racing distance for C:C horses was 6.2 ± 0.8 f, for C:T horses was 9.1 ± 2.4 f and for T:T horses was 10.5 ± 2.7 f. Furthermore, C:C horses have significantly greater muscle mass than T:T horses at two-years-old.

Skeletal muscle phenotypes clearly play a role in distinguishing distance aptitude, and there is a strong effect of *MSTN *genotype on distance [6]. However, heretofore, the effects of additional nuclear gene variants that may contribute to equine performance-related phenotypes have not been investigated. Therefore, we performed a genome-wide SNP-association study using the EquineSNP50 Bead Chip genotyping array in a cohort of elite race winning Thoroughbred horses. Animals were separated into two distinct phenotypic cohorts comprising short distance (≤ 8 f) and middle-long distance (> 8 f) race winners and genetic associations were evaluated using best race distance as a quantitative phenotype. This study was designed to identify additional genetic loci as indicators of race distance aptitude and to establish whether variation at the g.66493737C>T SNP was associated with inter-locus epistatic effects for race distance performance.

## Methods

### Ethics

This work has been approved by the University College Dublin, Ireland, Animal Research Ethics Committee.

### Study animals and cohorts

A repository of registered Thoroughbred horse blood or hair samples (*n *> 1,400) was collected from stud farms, racing yards and sales establishments in Ireland, Great Britain and New Zealand during 1997 to 2008. Each sample was categorized based on retrospective racecourse performance records. Only horses with performance records in Flat races were included in the study. The study cohort comprised elite Thoroughbreds that had won at least one Group race (Group 1, Group 2 or Group 3) or a Listed race--the highest standard and most valuable elite Flat races are known as Group (Stakes) races and Listed races are the next in status. Only elite race winning horses were included as elite races are most likely to reflect the truest test for distance. Race records were derived from three sources [Europe race records: The Racing Post on-line database http://www.racingpost.co.uk; Australasia and South East Asia race records: Arion Pedigrees http://www.arion.co.nz; North America race records: Pedigree Online Thoroughbred database http://www.pedigreequery.com].

Each sample was assigned a best race distance which was defined as the distance (furlongs, f) of the highest grade of race won [note: 1 furlong = 1/8 mile = 201.2 meters]. When multiple races of the same grade were won, then the distance of the most valuable race, in terms of prize money, was used. A set of elite Thoroughbred samples (*n *= 118) was selected from the repository, mostly comprising samples procured in Ireland and Great Britain (*i.e*. *n *= 5 samples [*n *= 3 ≤ 8 f, *n *= 2 > 8 f] were collected in New Zealand); though some had won their best race in North America. Animals with excessive consanguinity (within two generations) were avoided and over-representation of popular sires within the pedigrees was minimized as far as possible. One hundred and seven sires were represented in the total sample set. Genomic DNA was extracted from either fresh whole blood or hair samples using a modified version of a standard phenol/chloroform method [12] or the Maxwell 16 automated DNA purification system (Promega, WI, USA). DNA samples were quantified using Quant-iT PicoGreen dsDNA kits (Invitrogen, Carlsbad, CA) according to the manufacturer's instructions and the DNA concentrations were adjusted to 20 ng/μl.

For the case-control investigation we compared two cohorts: samples were subdivided into short (≤ 8 f, *n *= 68) and middle-long (> 8 f, *n *= 50) distance elite race winning cohorts (Table 1).

**Table 1:** Description of phenotype cohorts

	*N*	No. sires	Mean RPR	Range RPR	Mean BRD	Range BRD
All TBs	118	107	116	84-138	8.6	5-16
Short (≤ 8 f)	68	63	114	84-129	6.8	5-8
Middle-long (> 8 f)	50	48	120	107-138	11.3	9-16

All TBs (Thoroughbreds) were used for the quantitative association test analysis. Racing Post Ratings (RPR) represent handicap ratings (best lifetime RPR) that are indicative of performance ability. Best race distance (BRD) was the distance (f) of the highest grade of race (Group 1, 2, 3, Listed) won.

### Genotyping and quality control

Samples were genotyped using EquineSNP50 Genotyping BeadChips (Illumina, San Diego, CA). This array contains approximately 54,000 SNPs ascertained from the EquCab2 SNP database of the horse genome [13] and has an average density of one SNP per 43.2 kb. Genotyping was performed by AROS Applied Biotechnology AS, Denmark. The samples that were genotyped for this study were a subset of *n *= 187 samples genotyped in two separate batches (Batch 1, *n *= 96; Batch 2, *n *= 91). We included four pairs of duplicate samples in Batch 2 for QC purposes and observed greater than 99.9% concordance in the four pairs. In total, we successfully genotyped 53,795 loci. All samples had a genotyping rate of greater than 90%. We omitted SNPs which had a genotyping completion rate of less than 90%, were monomorphic or had minor allele frequencies (MAF) less than 5% in our samples from further analysis. We omitted 12,818 SNPs leaving 40,977 SNPs in our working build of the data and the overall genotype completion rate was 99.8%.

### Statistical analyses

All statistical analyses, including tests of association were performed using PLINK Version 1.05 [14]. We compared genotype frequencies in short and middle-long distance cohorts, testing for trait association using χ^2 ^tests with two degrees of freedom. To test for population stratification, the pairwise identity-by-state (IBS) distance was calculated for all individuals. A permutation test was performed to investigate IBS differences among the short and middle-long distance cohorts. The linear regression model was used to evaluate quantitative trait association using best race distance (f) as the phenotype. We report uncorrected *P*-values (*P*_unadj._) and *P*-values following correction for multiple testing using the Bonferroni method (*P*_Bonf._). Manhattan and Q-Q plots were generated in R using a modified version of code. The regional association plot was generated in R using a modified version of code available at http://www.broadinstitute.org.

Cohort-based association (short *vs *middle-long distance) and quantitative trait association tests were also performed for the g.66493737C>T SNP [6] and a novel 5'UTR *MSTN *SINE insertion (Ins227bp) polymorphism identified in this study. In addition, an analysis of genome-wide epistasis was performed in which the g.66493737C>T SNP was tested against all SNPs on the EquineSNP50 Genotyping BeadChip for epistatic interactions influencing best race distance. This test involved a linear regression analysis to investigate whether gene by gene interactions had a significant influence on best race distance. Linkage disequilibrium (LD) between g.66493737C>T and Ins227bp and between g.66493737C>T and all chromosome 18 SNPs on the EquineSNP50 Genotyping BeadChip was quantified as *r*^2^. A visual representation of haplotype blocks across a 1.7 Mb region on chromosome 18 was generated using Haploview [15,16].

### Re-sequencing *MSTN *flanking sequences

PCR primers were designed to cover ~2 kb of the 5'UTR and ~2 kb of the 3' UTR of *MSTN *genomic sequence using the PCR Suite extension to the Primer3 web-based primer design tool [17,18] (Table S1). Fifteen unrelated Thoroughbred DNA samples (g.66493737C>T, *n *= 5 C:C; *n *= 5 C:T, *n *= 5 T:T) were included in a re-sequencing panel to identify novel sequence variants. Bidirectional DNA sequencing of PCR products was performed by Macrogen Inc. (Seoul, Korea) using AB 3730xl sequencers (Applied Biosystems, Foster City, CA). Sequence variants were detected by visual examination of sequences following alignment using Consed version 19.0 [19].

### Bioinformatics

The software tool MatInspector [20] was used to search for transcription factor binding site consensus sequences present in 300 bp of the *MSTN *5' UTR region in which a novel SINE insertion (Ins227bp) polymorphism was detected. To investigate possible microRNA (miRNA) regulation of *MSTN *gene expression we screened the equine *MSTN *gene and flanking sequences for putative miRNA binding sites. A list of 407 predicted equine miRNAs [21] were inputted into the online tool DIANA microtest http://diana.pcbi.upenn.edu/cgi-bin/micro_t.cgi and a 14.7 kb segment containing the equine *MSTN *gene and ~5 kb of upstream and downstream sequence was inputted as the target sequence. SNPInspector [20] was used to investigate transcription factor binding sites at the g.66493737C>T locus.

### Genotyping the Chr18g.66495327Ins227bp66495326 (Ins227bp) polymorphism

A PCR-based assay for allele size discrimination was used to genotype the Ins227bp polymorphism in *n *= 165 samples. The following primers were used: forward 5'-ATCAGCTCACCCTTGACTGTAAC-3' and reverse 5'-TCATCTCTCTGGACATCGTACTG-3'. Alleles were determined as follows: Normal allele - 600 bp; and Insertion227bp allele - 827 bp.

## Results

### Genome-wide SNP-association study

**Figure 1:**
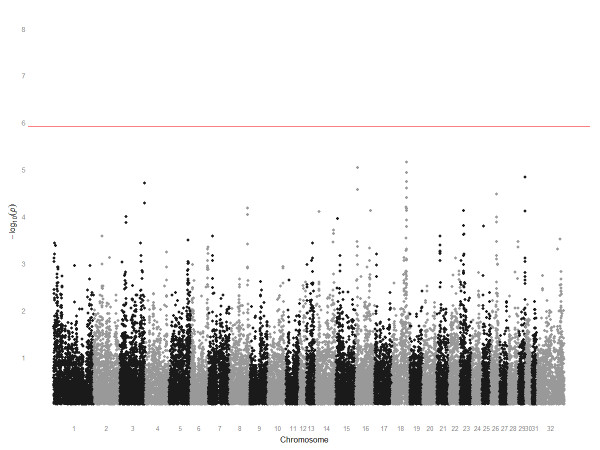
**Manhattan plot of *P*-value for genotype-phenotype GWAS in short **(≤ 8 f) **and middle-long **(> 8 f) **distance elite race winners**. The y-axis plots -log_10_(*P*-values) and the x-axis plots the physical position of the SNPs sorted by chromosome and chromosome position. The most significant SNP was on chromosome 18 (BIEC2-417495). No SNP remained statistically significant following correction for multiple-testing.

In a cohort-based genotype-phenotype investigation we compared two cohorts: short (≤ 8 f) and middle-long (> 8 f) distance elite race winners. The GWAS results, sorted by chromosome, are shown in Figure 1. The most significant SNP was on chromosome 18 (BIEC2-417495, *P*_unadj. _= 6.96 × 10^-6^) and five of the top ten SNPs were located together spanning a 2.4 Mb region on chromosome 18 (chr18:64725066-67186093). However, no SNP in this analysis reached genome-wide significance following correction for multiple-testing.

Pairwise IBS values were used to investigate population stratification between the short and middle-long cohorts. While on average phenotypically concordant pairs of individuals were more similar than phenotypically discordant pairs (*P *= 0.034), the overall difference between the two groups was negligible (< 0.0002).

**Figure 2:**
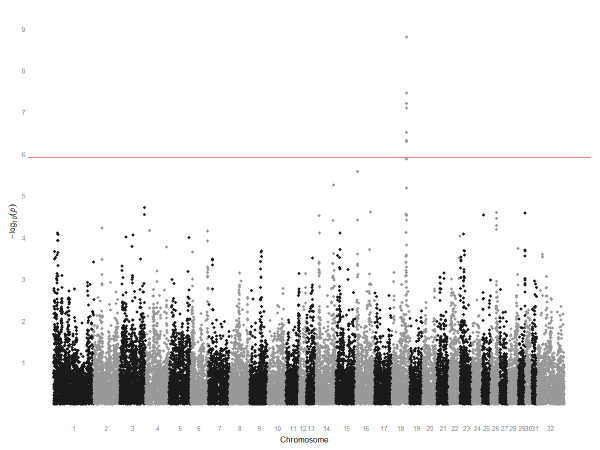
**Manhattan plot of *P*-value for quantitative trait GWAS using best race distance as phenotype**. The y-axis plots -log_10_(*P*-values) and the x-axis plots the physical position of the SNPs sorted by chromosome and chromosome position. A peak of association on chromosome 18 (chr18:65809482-67545806) encompassed a ~1.7 Mb region (shown in Figure 3). Seven of the chromosome 18 SNPs remained significant following correction for multiple testing. The most significant SNP was BIEC2-417495 (*P*_Bonf. _= 6.58 × 10^-5^).

**Figure 3:**
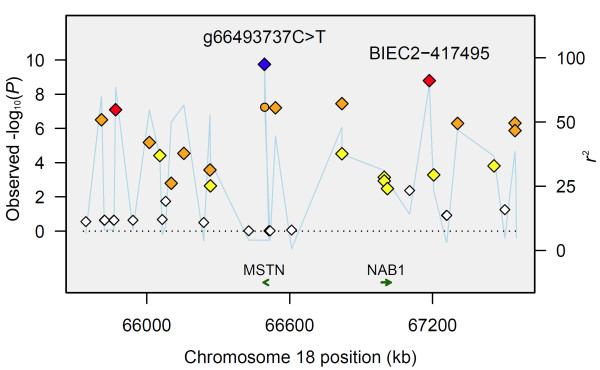
**A regional plot for the 1.8 Mb peak of association on chromosome 18 containing the *MSTN *and *NAB1 *genes**. Association plot of the 1.8 Mb region encompassing 40 SNPs (diamonds) and the Ins227bp polymorphism (circle) ranging from one SNP upstream and one SNP downstream of the seven SNPs significantly associated with optimum racing distance following correction for multiple testing. The y-axes plot -log_10_(*P*-values) for each SNP (diamonds) and *r*^2 ^(blue line) between g.66493737C>T and all other SNPs. The x-axis plots the physical position of each SNP in the region. The best SNP, g.66493737C>T, is indicated with a blue diamond. Each SNP is color coded according to the strength of LD with g.66493737C>T: *r*^2 ^≥ 0.8, red; *r*^2 ^≥ 0.5 < 0.8, orange; *r*^2 ^≥ 0.2 < 0.5, yellow; *r*^2 ^< 0.2, white.

Using the linear regression model, we considered best race distance as a quantitative phenotype and observed the same peak of association on chromosome 18 (chr18:65809482-67545806) (Figure 2; Additional File 1). The top eight SNPs encompassed a 1.7 Mb region on chromosome 18 (Figure 3) and seven reached genome-wide significance following correction for multiple testing (*P*_Bonf. _< 0.05). The most significant SNP was also the most significant in the cohort-based analysis: BIEC2-417495 (*P*_unadj. _= 1.61 × 10^-9^; *P*_Bonf. _= 6.58 × 10^-5^).

### Candidate performance-associated genes

We investigated candidate genes in the 1.7 Mb (Chr18:65809482-67545806) region on chromosome 18 that encompassed the seven SNPs that reached genome-wide significance. Eleven protein coding genes were identified, including the myostatin gene (*MSTN*) and the NGFI-A binding protein 1 (EGR1 binding protein 1) gene (*NAB1*).

### Polymorphism detection in equine *MSTN *flanking sequences

We previously identified SNPs in intron 1 of the equine *MSTN *gene by re-sequencing the coding and intronic sequence [6]. However, genomic sequence or structural variation in the flanking regions was not investigated. Therefore, for the present study we re-sequenced 2,251 bp (chr18:66494683-66496834) of the 5' UTR and 2,155 bp (chr18:66488052-66490207) of the 3' UTR of the *MSTN *gene (Additional File 2) and identified four novel SNPs in the 3' UTR and a SINE insertion polymorphism in the 5' UTR. An overview of sequence and structural variation in the equine *MSTN *gene and flanking sequences is provided in Additional File 3.

Polymorphisms in the 3' UTR of the *MSTN *gene have been associated with muscle hypertrophy in sheep and are considered likely to function via creation of *de novo *target sites for the microRNAs (miRNA) miR-1 and miR-206 [22]. Therefore, using a set of equine miRNAs (*n *= 407) described by Zhou and colleagues [21] we investigated the presence of putative miRNA binding sites within ~5 kb upstream and downstream flanking sequences of the *MSTN *gene. Five putative miRNA binding sites were identified, though none was polymorphic: *i.e*. no putative miRNA binding site was associated with any of the eight SNP alleles.

Following re-sequencing in the 5' UTR region of the *MSTN *gene, we identified a 227 bp insertion polymorphism at chr18:66495327-[Insertion227bp]-66495326 (henceforth referred to as Ins227bp), located 146 bp from the start of exon 1 (Exon1Start: 66495180). The insertion sequence is as follows:

GGGGCTGGCCCCGTGGCCGAGTGGTTAAGTTCGTGCGCTCCGCTGCAGGCGGCCCAGTGTTTCGTCGGTTCGAGTCCTGGGCGCGGACATGGCACTG

CTCGTCGGACCACGCTGAGGCAGCGTCCCACATGCCACAACTAGAGGAACCCACAACGAAGAATACACAACTATGTACCGGGGGGCTTTGGGGAGAA

AAAGGAAAATAAAATCTTTAAAAAGCCACTTGG.

A BLAST search identified the insertion sequence as a horse-specific repetitive DNA sequence element (SINE) known as ERE-1 [23]. Also, MatInspector analysis indicated that the insertion may disrupt an E-box motif.

In 14 of the 15 sequenced samples, the Ins227bp allele was in concordance with the C-allele at g.66493737C>T. As complete concordance was not observed, we genotyped a set of *n *= 165 samples to determine the extent of concordance between the Ins227bp and g.66493737C>T polymorphisms. We performed parallel association tests for the same set of samples to evaluate the relative performance of the two polymorphisms as predictors of optimum racing distance. The g.66493737C>T SNP performed better in an association test with best race distance (*P *= 5.24 × 10^-13^) than the Ins227bp polymorphism (*P *= 5.54 × 10^-10^). Analysis of the sequence surrounding g.66493737C>T indicated that alternate alleles may result in the gain of a putative Homeobox C8/Hox-3alpha transcription factor binding site and/or the disruption of putative Distal-less homeobox 3, E2F and Pdx1 transcription factor binding sites.

### Linkage disequilibrium

**Figure 4:**
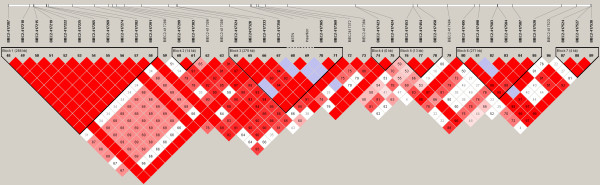
**A visual representation of haplotype blocks across a 1.7 Mb region on chromosome 18.** The g.66493737C>T SNP was included in block 3;  BIEC2-417495 was included in block 6.

Pairwise tests of linkage disequilibrium (LD) were performed between g.66493737C>T and Ins227bp, and between g.66493737C>T and the 1,373 chromosome 18 SNPs represented on the genotyping array. LD was highest between g.66493737C>T and BIEC2-417495 (*r*^2 ^= 0.86). LD between g.66493737C>T and Ins227bp was *r*^2 ^= 0.73. Seven discrete haplotype blocks were identified in the 1.7 Mb peak of association on chromosome 18. The g.66493737C>T SNP was included in block 3; BIEC2-417495 was included in block 6 (Figure 4).

## Discussion

We have previously described an association between optimum racing distance and a SNP (g.66493737C>T) in the equine *MSTN *gene in Thoroughbred Flat racehorses [6]. Candidate gene approaches are designed considering *a priori *hypotheses and do not allow the opportunity for evaluation of the effect of the gene in the context of the entire genome, nor do they allow for the identification of other genes contributing to the phenotype [24,25]. Therefore, employing a hypothesis-free approach we investigated genome-wide influences on optimum racing distance by conducting a genome-wide SNP-association study in a cohort of elite Thoroughbred racehorses.

The genomic region on chromosome 18 containing the *MSTN *gene was the highest ranked region in the GWAS for best racing distance, reaching genome-wide significance for a set of seven SNPs within a 1.7 Mb region. The best SNP (BIEC2-417495) and the second best SNP (BIEC2-417372) were 692 kb and 28 kb from the *MSTN *gene, respectively. We searched the region for other plausible candidate genes and identified the NGFI-A binding protein 1 (EGR1 binding protein 1) gene (*NAB1*) located ~170 kb from BIEC2-417495. The product of the *NAB1 *gene is highly expressed in cardiac muscle and has been reported to be a transcriptional regulator of cardiac growth [26]. Its principal role is in its interaction with the early growth response 1 (EGR-1) transcriptional activator that is involved in regulation of cellular growth and differentiation [27].

We considered *NAB1 *as a strong candidate gene to influence an athletic performance phenotype as we have previously identified *EGR-1 *mRNA transcript alterations (+1.6-fold, *P = *0.014) in skeletal muscle immediately following a bout of treadmill exercise in untrained Thoroughbred horses [28]. Twelve SNPs located within the *NAB1 *genomic sequence (chr18:g.66995249-67021729) are documented in the EquCab2 SNP database, and three are contained on the EquineSNP50 Genotyping BeadChip. After correction for multiple testing, there were no detectable associations between the three *NAB1 *SNPs and the trait (BIEC2-417453, P_unadj. _= 0.0007, rank 144; BIEC2-417454, P_unadj. _= 0.0012, rank 210; and BIEC2-417458, P_unadj. _= 0.0032, rank 421). Therefore, we did not further consider *NAB1 *as a potential major contributor to variation in optimum racing distance.

Results from analyses of gene expression generated since our initial report of an association between *MSTN *genomic variation and optimum racing distance in Thoroughbreds support the hypothesis that the *MSTN *gene is functionally relevant to racing performance variation. In a transcriptome-wide investigation using digital gene expression (DGE) technology, we identified the greatest alteration in mRNA abundance in transcripts from *MSTN *in Thoroughbred skeletal muscle following a ten-month period of exercise training. Seventy-four annotated transcripts were differentially expressed between pre-and post-training states and among the 58 genes with decreased expression, *MSTN *mRNA transcripts were the most significantly reduced (-4.2-fold, *P *= 0.0043) [29].

Consequently, we focused on comprehensively evaluating variation in the *MSTN *gene by re-sequencing ~2 kb of the 3' and 5' flanking sequences. Four novel 3' UTR SNPs and a 227 bp SINE insertion (Ins227bp) polymorphism located 146 bp upstream of the coding region start site were identified. We investigated whether the 3' UTR SNPs may abrogate existing or create *de novo *putative miRNA binding sites, as has been described for *MSTN *influenced phenotypic variation in Texel sheep [22]. However, there was no evidence for alterations in putative miRNA binding sites. Next, because of the close proximity to the transcriptional start site, we considered the Ins227bp polymorphism as a strong functional candidate contributing to variation in racing performance. However, a comparative evaluation of association using the same set of samples (*n *= 165) demonstrated that the g.66493737C>T SNP displayed a stronger association (*P = *5.24 × 10^-13^) with best race distance than the Ins227bp polymorphism (*P *= 5.54 × 10^-10^).

An evaluation of LD showed that the strongest association was between g.66493737C>T and the most significant SNP in the present study, BIEC2-417495. A comparison of trait association in the same set of samples (*n *= 118) confirmed the superior power of the g.66493737C>T SNP (*P = *1.02 × 10^-10^) in the prediction of best race distance when compared with BIEC2-417495 (*P*_unadj. _= 1.61 × 10^-9^). The significance values and genotype frequencies for the top SNPs in the GWAS and the g.66493737C>T SNP are shown in Table 2. In addition, we investigated whether g.66493737C>T may interact with other SNPs represented on the EquineSNP50 genotyping array; however, no significant interaction was observed to influence best race distance (*P *> 0.0001 for all interactions). Therefore, the effect of genotype on racing phenotype is highly likely a result of the previously reported variation in the *MSTN *gene at locus g.66493737C>T.

**Table 2:** Significance values (unadjusted and Bonferroni corrected *P *values) for the top SNPs associated with optimum race distance.

CHR	SNP	UNADJ P	BONF. P	A1	A2	A11	A12	A22
18	g.66493737C>T	1.02E-10	N/A	T	C	0.1538	0.5962	0.2500
18	BIEC2-417495	1.61E-09	6.58E-05	T	C	0.1709	0.5983	0.2308
18	BIEC2-417423	3.55E-08	0.001454	G	A	0.1017	0.5169	0.3814
18	BIEC2-417372	6.21E-08	0.002545	G	A	0.0932	0.5424	0.3644
18	BIEC2-417274	8.08E-08	0.003312	T	G	0.1864	0.6017	0.2119
18	BIEC2-417210	3.13E-07	0.01281	C	T	0.2119	0.5763	0.2119
18	BIEC2-417524	4.87E-07	0.01995	G	A	0.1186	0.5763	0.3051
18	BIEC2-417507	5.09E-07	0.02086	C	A	0.1368	0.5897	0.2735

A11: genotype frequency for homozygotes (allele 1) in the population (n = 118); A12: genotype frequency for heterozygotes; A22 genotype frequency for homozygotes (allele 2). Correction for multiple testing was not applied for g.66493737C>T; however, the association remains stronger (*P*_Bonf. _= *4.18 *× 10^-6^*) *after application of a correction factor.

It is important to note that the sample size used for the present study is relatively small. However, the results of the quantitative trait GWAS demonstrate that the sample size used was sufficient to detect a major genetic effect such as that manifested at the *MSTN *locus. A lower sample size requirement for GWAS in the Thoroughbred is supported by population genomics analyses of this population in comparison to other horse breeds. These demonstrate that the extent of LD in the Thoroughbred is significantly greater than that measured in other horse populations, being comparable to LD estimates in inbred dog breeds [13].The high LD in Thoroughbreds is a reflection of low effective population size, which enables detection of associations with smaller sample sizes.

The mechanism by which the g.66493737C>T sequence variant may affect the muscle phenotype in horses is not clear; however we propose a direct effect of the SNP on the control of myocyte development. Myostatin is a growth and differentiation factor (GDF8) that functions as a negative regulator of skeletal muscle mass development and results in hypertrophied muscle phenotypes in a range of mammalian species, including horse. Consistent with this role myostatin has been shown to repress the proliferation and differentiation of cultured myocytes [30-32]. The proliferation of myoblasts is determined by the control and progression of the cell cycle, a role which has been assigned to members of the E2F family of transcription factors [33]. The g.66493737C>T SNP is located within the sequence of a putative E2F transcription factor binding site in intron 1 of the *MSTN *gene. It may therefore be plausible to propose a mechanism by which allele-specific binding of E2F to myostatin influences the growth and development of myocytes following signalling from upstream effector proteins such as retinoblastoma protein [34]. Genotype-specific gene expression studies will shed light on the allele-specific effect on function.

## Conclusion

This study represents the first genome-wide investigation of sequence variant association with an athletic performance phenotype in Thoroughbred racehorses. It complements a recent DGE transcriptome-wide investigation of functional responses to exercise training in Thoroughbred skeletal muscle, which identified *MSTN *mRNA transcripts as the most significantly altered following a ten month period of training. The present study provides clear evidence that the previously reported polymorphism in equine *MSTN *at locus g.66493737C>T is the most powerful genome-wide predictor of optimum racing distance in Thoroughbred horses.

## Competing interests

The research has been funded by Equinome Ltd. DEM and EWH are shareholders in Equinome Ltd. Equinome Ltd. has been granted a licence for commercial use of the data which is contained within patent applications: United States Provisional Serial Number 61/136553; Irish patent application number 2008/0735 and 2010/0151; Patent Cooperation Treaty number PCT/IE2009/000062. The following authors are named on the applications: EWH, JG, and DEM.

## Authors' contributions

EWH initiated, designed and coordinated the study, collected samples, determined cohorts and wrote the manuscript. BAM conducted statistical analyses and prepared the figures. JG prepared DNA for genotyping, performed re-sequencing and genotyping and assisted with manuscript preparation. RW assisted with re-sequencing and genotyping. DEM designed the re-sequencing study, contributed to project organization and assisted manuscript preparation. All authors have read and approved the final manuscript.

## Supplementary Material

Additional file 1 **Quantitative association test results for best race distance**. Unadjusted and FDR corrected *P*-values are given. Click here for file

Additional file 2 **PCR and sequencing primers for re-sequencing MSTN flanking regions**. Primers were designed to cover ~2 kb of the 3' UTR and ~2 kb of the 5' UTR of the *MSTN *gene. Click here for file

Additional file 3 **Sequence and structural variation in the coding, intronic and flanking sequences of the equine *MSTN *gene**. Sequences are provided for the + strand. Click here for file

## References

[B1] Willett P The classic racehorse 1981 London: Stanley Paul

[B2] Williamson SA Beilharz RG The inheritance of speed, stamina and other racing performance characters in the Australian Thoroughbred J Anim Breed Genet 1998 115 1 1 16

[B3] Barrey E Valette JP Jouglin M Blouin C Langlois B Heritability of percentage of fast myosin heavy chains in skeletal muscles and relationship with performance Equine Vet J Suppl 1999 30 289 292 10659270 10.1111/j.2042-3306.1999.tb05236.x

[B4] Rivero JL Serrano AL Henckel P Aguera E Muscle fiber type composition and fiber size in successfully and unsuccessfully endurance-raced horses J Appl Physiol 1993 75 4 1758 1766 8282629 10.1152/jappl.1993.75.4.1758

[B5] Rivero J-L Piercy RJ Hinchcliff KW, Kaneps AJ, Geor RJ Muscle physiology: responses to exercise and training Equine exercise physiology: the science of exercise in the athletic horse 2008 ix Edinburgh: Elsevier Saunders 463

[B6] Hill EW Gu J Eivers SS Fonseca RG McGivney BA Govindarajan P Orr N Katz LM MacHugh DE A sequence polymorphism in *MSTN *predicts sprinting ability and racing stamina in thoroughbred horses PLoS ONE 2010 5 1 e8645 10.1371/journal.pone.0008645 20098749 PMC2808334

[B7] Grobet L Martin LJ Poncelet D Pirottin D Brouwers B Riquet J Schoeberlein A Dunner S Menissier F Massabanda J A deletion in the bovine myostatin gene causes the double-muscled phenotype in cattle Nat Genet 1997 17 1 71 74 10.1038/ng0997-71 9288100

[B8] McPherron AC Lawler AM Lee SJ Regulation of skeletal muscle mass in mice by a new TGF-beta superfamily member Nature 1997 387 6628 83 90 10.1038/387083a0 9139826

[B9] McPherron AC Lee SJ Double muscling in cattle due to mutations in the myostatin gene Proc Natl Acad Sci USA 1997 94 23 12457 12461 10.1073/pnas.94.23.12457 9356471 PMC24998

[B10] Mosher DS Quignon P Bustamante CD Sutter NB Mellersh CS Parker HG Ostrander EA A mutation in the myostatin gene increases muscle mass and enhances racing performance in heterozygote dogs PLoS Genet 2007 3 5 e79 10.1371/journal.pgen.0030079 17530926 PMC1877876

[B11] Schuelke M Wagner KR Stolz LE Hubner C Riebel T Komen W Braun T Tobin JF Lee SJ Myostatin mutation associated with gross muscle hypertrophy in a child N Engl J Med 2004 350 26 2682 2688 10.1056/NEJMoa040933 15215484

[B12] Sambrook J Russell DW Molecular cloning: a laboratory manual 2001 3 Cold Spring Harbor, N.Y.: Cold Spring Harbor Laboratory Press

[B13] Wade CM Giulotto E Sigurdsson S Zoli M Gnerre S Imsland F Lear TL Adelson DL Bailey E Bellone RR Genome sequence, comparative analysis, and population genetics of the domestic horse Science 2009 326 5954 865 867 10.1126/science.1178158 19892987 PMC3785132

[B14] Purcell S Neale B Todd-Brown K Thomas L Ferreira MA Bender D Maller J Sklar P de Bakker PI Daly MJ PLINK: a tool set for whole-genome association and population-based linkage analyses Am J Hum Genet 2007 81 3 559 575 10.1086/519795 17701901 PMC1950838

[B15] Barrett JC Haploview: Visualization and analysis of SNP genotype data Cold Spring Harb Protoc 2009 2009 10 pdb ip71 20147036 10.1101/pdb.ip71

[B16] Barrett JC Fry B Maller J Daly MJ Haploview: analysis and visualization of LD and haplotype maps Bioinformatics 2005 21 2 263 265 10.1093/bioinformatics/bth457 15297300

[B17] Rozen S Skaletsky H Primer3 on the WWW for general users and for biologist programmers Methods Mol Biol 2000 132 365 386 10547847 10.1385/1-59259-192-2:365

[B18] van Baren MJ Heutink P The PCR suite Bioinformatics 2004 20 4 591 593 10.1093/bioinformatics/btg473 14751986

[B19] Gordon D Abajian C Green P Consed: a graphical tool for sequence finishing Genome Res 1998 8 3 195 202 9521923 10.1101/gr.8.3.195

[B20] Cartharius K Frech K Grote K Klocke B Haltmeier M Klingenhoff A Frisch M Bayerlein M Werner T MatInspector and beyond: promoter analysis based on transcription factor binding sites Bioinformatics 2005 21 13 2933 2942 10.1093/bioinformatics/bti473 15860560

[B21] Zhou M Wang Q Sun J Li X Xu L Yang H Shi H Ning S Chen L Li Y In silico detection and characteristics of novel microRNA genes in the *Equus caballus *genome using an integrated *ab initio *and comparative genomic approach Genomics 2009 94 2 125 131 10.1016/j.ygeno.2009.04.006 19406225

[B22] Clop A Marcq F Takeda H Pirottin D Tordoir X Bibe B Bouix J Caiment F Elsen JM Eychenne F A mutation creating a potential illegitimate microRNA target site in the myostatin gene affects muscularity in sheep Nat Genet 2006 38 7 813 818 10.1038/ng1810 16751773

[B23] Sakagami M Ohshima K Mukoyama H Yasue H Okada N A novel tRNA species as an origin of short interspersed repetitive elements (SINEs). Equine SINEs may have originated from tRNA(Ser) J Mol Biol 1994 239 5 731 735 10.1006/jmbi.1994.1410 8014993

[B24] Jorgensen TJ Ruczinski I Kessing B Smith MW Shugart YY Alberg AJ Hypothesis-driven candidate gene association studies: practical design and analytical considerations Am J Epidemiol 2009 170 8 986 993 10.1093/aje/kwp242 19762372 PMC2765367

[B25] Tabor HK Risch NJ Myers RM Opinion: Candidate-gene approaches for studying complex genetic traits: practical considerations Nat Rev Genet 2002 3 5 391 397 10.1038/nrg796 11988764

[B26] Buitrago M Lorenz K Maass AH Oberdorf-Maass S Keller U Schmitteckert EM Ivashchenko Y Lohse MJ Engelhardt S The transcriptional repressor Nab1 is a specific regulator of pathological cardiac hypertrophy Nat Med 2005 11 8 837 844 10.1038/nm1272 16025126

[B27] Thiel G Kaufmann K Magin A Lietz M Bach K Cramer M The human transcriptional repressor protein NAB1: expression and biological activity Biochim Biophys Acta 2000 1493 3 289 301 11018254 10.1016/s0167-4781(00)00207-4

[B28] McGivney BA Eivers SS MacHugh DE MacLeod JN O'Gorman GM Park SD Katz LM Hill EW Transcriptional adaptations following exercise in thoroughbred horse skeletal muscle highlights molecular mechanisms that lead to muscle hypertrophy BMC Genomics 2009 10 638 10.1186/1471-2164-10-638 20042072 PMC2812474

[B29] McGivney BA McGettigan PA Browne JA Evans AC Fonseca RG Loftus BJ Lohan A MacHugh DE Murphy BA Katz LM Characterization of the equine skeletal muscle transcriptome identifies novel functional responses to exercise training BMC Genomics 2010 11 398 10.1186/1471-2164-11-398 20573200 PMC2900271

[B30] Joulia D Bernardi H Garandel V Rabenoelina F Vernus B Cabello G Mechanisms involved in the inhibition of myoblast proliferation and differentiation by myostatin Exp Cell Res 2003 286 2 263 275 10.1016/S0014-4827(03)00074-0 12749855

[B31] Langley B Thomas M Bishop A Sharma M Gilmour S Kambadur R Myostatin inhibits myoblast differentiation by down-regulating MyoD expression J Biol Chem 2002 277 51 49831 49840 10.1074/jbc.M204291200 12244043

[B32] Thomas M Langley B Berry C Sharma M Kirk S Bass J Kambadur R Myostatin, a negative regulator of muscle growth, functions by inhibiting myoblast proliferation J Biol Chem 2000 275 51 40235 40243 10.1074/jbc.M004356200 10976104

[B33] Polager S Ginsberg D p53 and E2f: partners in life and death Nat Rev Cancer 2009 9 10 738 748 10.1038/nrc2718 19776743

[B34] Hallstrom TC Nevins JR Balancing the decision of cell proliferation and cell fate Cell Cycle 2009 8 4 532 535 19182518 10.4161/cc.8.4.7609 PMC3018328

